# Colonic Barrier Dysfunction and Inflammation Induced by Nano‐/Micro‐Plastics Fibers in Mice via the FAK/NF‐κB/iNOS and TLR4/NF‐κB/iNOS Pathways

**DOI:** 10.1002/jat.70196

**Published:** 2026-04-01

**Authors:** Yuting Li, Shibo Bao, Puyang Huang, Jiahui Wang, Hongfeng Li, Yahong Liu, Kai Wang, Jianjun Du, Lingjun Yan, Guowei Pan, Wei Sun

**Affiliations:** ^1^ Key Laboratory of Environmental Stress and Chronic Disease Control & Prevention (China Medical University), Ministry of Education Shenyang China; ^2^ Research Center for Universal Health, School of Public Health, China Medical University Shenyang China; ^3^ Liaoning Provincial Key Laboratory of Early Warning, Intervention Technology and Countermeasure Research for Major Public Health Events Shenyang China; ^4^ Key Laboratory of Electromagnetic Processing of Materials (Ministry of Education) Northeastern University Shenyang China; ^5^ Department of Retirement Affairs the First Hospital of China Medical University Shenyang China; ^6^ State Key Laboratory of Fine Chemicals, Frontiers Science Center for Smart Materials, Dalian University of Technology Dalian China

**Keywords:** colonic barrier, FAK/NF‐κB/Inos, fibers and beads, polystyrene microplastics, TLR4/NF‐κB/iNOS

## Abstract

As emerging environmental contaminants, nano‐ and micro‐plastics (NMPs) have drawn increasing research attention due to potential hazards to organisms. Particle shape is a key factor influencing the toxic effects of NMPs, and NMPs of different shapes cause varying degrees of tissue damage. We investigated the specific mechanisms of polystyrene NMPs (PS‐NMPs) with different shapes underlying damage to the colonic intestinal barrier. We divided 120 five‐week‐old male C57BL/6J mice into seven groups that were orally exposed to different doses of PS‐NMPs for 5 weeks. Hematoxylin and eosin staining showed that both shapes of PS‐NMPs induced colon pathological injuries, with more pronounced damage in the PS‐Fibers groups. DAO and D‐LA levels were increased in the medium‐ and high‐dose groups. In all PS‐Fibers groups, DAO and D‐LA levels were significantly elevated. Immunohistochemical analysis revealed discontinuous distribution and reduced expression of ZO‐1 and occludin in the PS‐NMPs groups. Alcian blue‐periodic acid–Schiff staining results indicated that both types of PS‐NMPs significantly reduced the number of goblet cells. The levels of ZO‐1, occludin, MUC2, and E‐cadherin were significantly decreased in the high‐dose PS‐Beads group and all PS‐Fibers groups. The expression of pro‐inflammatory cytokines was significantly increased in the high‐dose PS‐Beads group and all PS‐Fibers groups. Mechanistically, PS‐Fibers induced colonic intestinal barrier damage by activating both the TLR4/NF‐κB/iNOS and FAK/NF‐κB/iNOS pathways, whereas PS‐Beads induced damage primarily via the TLR4/NF‐κB/iNOS pathway. These findings highlight the effect of differently shaped PS‐NMPs on affecting colonic barrier function, thereby providing new insights into the adverse health effects of NMPs.

## Abstract 2

Nano‐ and micro‐plastics (NMPs) pose emerging risks to human health. This study demonstrates that particle shape critically influences toxicity. Polystyrene fibers induced more severe colonic barrier damage than spherical particles in mice. Fibers caused pronounced epithelial injury, goblet cell loss, tight junction disruption, and inflammation via activation of TLR4/NF‐κB/iNOS and FAK/NF‐κB/iNOS pathways. These findings highlight shape‐dependent mechanisms underlying NMPs‐induced intestinal toxicity.

## 1 Introduction

As emerging environmental contaminants, nano‐ and micro‐plastics (NMPs) have drawn growing concern due to their ubiquitous distribution, persistence, and potential harmful effects on ecosystems and human health (Jung et al. 2022; Ramsperger et al. 2023). The term “microplastics” refers to plastic particles in the environment measuring between 1 μm and 5 mm, whereas “nanoplastics” are generally defined as having a size range from 1 to 100 nm (Anand et al. 2023; Ebrahimi et al. 2022). Based on shape, NMPs can be categorized as microbeads, fibers, granules, films, fragments, and foams (Kwon et al. 2020). Because of high mobility, resistance to degradation, and strong adsorption capacity (Liu, Hu, et al. 2022; Liang et al. 2022), NMPs pollution have become increasingly severe global environmental pollutants. Multiple studies have reported the presence of NMPs in human tissues, with fibers being the predominant form (Momeni et al. 2025; Amato‐Lourenco et al. 2024; Wang et al. 2023; Saenen et al. 2023). Human intake of NMPs has been estimated at 4.7 × 10^3^ particles per person per year, with the fibrous form accounting for more than 50% of NMPs isolated from various food products (Sobhani et al. 2020; Guo et al. 2020). Physicochemical characterization and quantitative size analysis have confirmed the presence of NMPs in postmortem human brain, kidney, and liver tissues (Amato‐Lourenco et al. 2024). Approximately 78%–83% NMPs identified in human tissues were primarily composed of elongated nanoscale fibers.

Both in vitro and in vivo studies have reported that the accumulation of NMPs in the gastrointestinal tract and the intestinal barrier was primarily affected by subsequent toxic effects, which include increased production of intestinal mucus, reduced numbers of intestinal mucins (Li et al. 2025), compositional alterations to the gut microbiome, and changes to the populations of intestinal immune cells (Chen et al. 2025). The intestinal epithelium serves as a physical barrier between the intestinal lumen and internal environment (Abdel‐Zaher et al. 2023). Exposure to polystyrene (PS)‐NMPs induces toxic effects by disrupting the physical, chemical, and immunological barriers of the colon, which results in shortening of colon length, exacerbation of histopathological damage, and inflammation (Luo et al. 2022). A prior study using an acute mouse exposure model, demonstrated that ingestion of PS‐NMPs induced toxic effects in mouse colon tissue (Djouina et al. 2022). In addition, ingestion of NMPs was reported to reduce colonic mucus secretion, enhance intestinal permeability, and increase levels of inflammatory cytokines (Chen et al. 2025; Djouina et al. 2022; Liu, Li, et al. 2022).

In environmental and biological samples, NMPs are predominantly fibrous (Osman et al. 2023). However, studies on the toxicity of fibrous NMPs remain limited compared to spherical NMPs. The mechanisms related to tissue damage caused by NMPs primarily involve oxidative stress, inflammation, mitochondrial dysfunction, and apoptosis (da Silva Brito et al. 2024; Gu et al. 2025; Song et al. 2024; Baroni et al. 2025; Seo et al. 2022). Inflammation is the most common protective mechanism triggered after NMPs enter colon tissues. Among the various proposed pathways, the nuclear factor‐κB (NF‐κB)/inducible nitric oxide synthase (iNOS) axis plays a crucial role in regulating colonic inflammation (Yang et al. 2025). NF‐κB, a central mediator of inflammatory responses, is bound to its inhibitory protein IκB in the cytoplasm under resting state conditions (Zhang et al. 2023). Upon cellular stimulation (e.g., oxidative stress and pathogen infection), the NF‐κB dimer is released and translocated into the nucleus, promoting the transcription of genes encoding pro‐inflammatory cytokines (TNF‐α, IL‐6 and IL‐1β), and adhesion molecules. As a key member of the NOS family, iNOS exerts a profound influence on colitis via the NF‐κB/iNOS pathway (Jin et al. 2024). A study of molecular‐level alterations to colitis‐related factors found that specific inhibition of the NF‐κB pathway targeted activation of the downstream inflammatory mediator iNOS in human colon adenocarcinoma Caco‐2 cells (Hunto et al. 2020). Various stimuli such as mechanical stress, environmental chemicals, and pathogen invasion (Alruhaimi et al. 2023; Feng et al. 2018; Chuang et al. 2021; Patra et al. 2016) can induce colonic inflammation. In response to inflammation, Toll‐like receptor 4 (TLR4) and focal adhesion kinase (FAK) activate downstream cascades (e.g., NF‐κB/iNOS) through distinct pathways, thereby promoting inflammation (Yang et al. 2025; Yang et al. 2023). Notably, PS‐NMPs were reported to alter the composition of the gut microbiota, disrupt intestinal barrier function, and enhance inflammatory responses through the TLR4/MyD88/NF‐κB signaling cascade (Yang et al. 2023).

Accordingly, we conducted toxicity studies using C57BL/6 mice that were intragastrically administered PS‐Fibers (length: 10 μm, diameter: 500 nm) and PS‐Beads (diameter: 500 nm) at varying concentrations to investigate the differential toxic effects on mouse colon tissue. Additionally, normal fetal human colonic (FHC) cells were treated separately with PS‐Fibers or PS‐Beads to explore the specific mechanisms employed by differently shaped PS‐NMPs to induce damage to the intestinal barrier in the colon.

## 2 Materials and Methods

### 2.1 Preparation of PS‐Fibers

PS powder (8–15 wt%, 1.85 g) and a trace amount (0.6 wt%) of benzyltriethylammonium chloride were dissolved in N, N‐dimethylformamide (10 mL) under continuous magnetic stirring for 24 h to generate a homogenously dissolved PS spinning solution. An electrospinning apparatus (Tongli Tech Co. Ltd., Shenzhen, China) was conditioned to an optimal temperature (18°C–23°C) and humidity (62%). Initial settings for the collector rotation speed (700 rpm) and solution feed rate (0.6 mL/min) were established. After the collector parameters were stabilized, the electrospinning voltage was calibrated to initiate fiber formation. The electrospinning process was performed with an electric field of 30 kV/cm, feed rate of 0.6 mL/h, and distance between the needle tip and collector of 25 cm. The uniformly aligned fibers were carefully detached from the aluminum foil and prepared for frozen sectioning.

Sliced PS‐Fibers (300 mg) were added to a beaker and mixed with ultrapure water (150 mL) in an oil‐bath heated at 70°C for 1 h under continuous agitation. The suspension was then centrifuged at 10,000 × g for 5 min to isolate solid residues. Heating in an oil‐bath and centrifugation were repeated three times to ensure thorough purification. The solid fibers were collected, dried in an oven, and weighed.

Batches of the dried fibers were magnetically stirred in a fixed volume of ultrapure water to optimize dispersion in dual‐vortex agitation mode at a constant rotational speed of 500 × g for 96 h. This rigorous protocol ensured that a standardized PS‐Fiber solution, with uniform dimensions and dispersion, was formed and met stringent criteria.

### 2.2 Characterization of the PS‐Fibers and PS‐Beads

The morphological structures of PS‐Fibers and PS‐Beads were characterized by field emission scanning electron microscopy (SEM) (Gemini 300, Carl Zeiss AG, Oberkochen, Germany). Prior to imaging, the samples were subjected to ultrasonic dispersion for 15 min to ensure homogeneity. After air‐drying under controlled dust‐free conditions, the samples were heated to 100°C on a hot plate for 5 min to remove residual moisture. For SEM observation, the dried samples were mounted on conductive adhesive tape and sputter‐coated with a thin gold film at a current of 10 mA. SEM was performed using a secondary electron detector at an accelerating voltage of 20 kV. The SEM results revealed that both PS‐Fibers of varying lengths and PS‐Beads exhibited highly uniform material characteristics along with the desired nano structural morphology. PS‐Beads were purchased from Tianjin BaseLine ChromTech Research Centre (6‐1‐0050).

### 2.3 Animals and Experimental Design

Healthy, specific pathogen‐free (SPF) male C57BL/6J mice were selected. The mice (*n* = 105; age, 5 weeks) were randomly assigned to one of seven groups (*n* = 15/group) using a random number table: negative control group, low‐dose PS‐Beads group (2.5 mg/kg), medium‐dose PS‐Beads group (25 mg/kg), high‐dose PS‐Beads group (50 mg/kg), low‐dose PS‐Fibers group (2.5 mg/kg), medium‐dose PS‐Fibers group (25 mg/kg), or high‐dose PS‐Fibers group (50 mg/kg). Globally, the estimated daily intake of plastic particles for humans (approximately 60 kg body weight) is approximately 0.23–11.9 mg/kg. The equivalent dose (mg/kg), based on body surface area was calculated as human dose (mg/kg) × [Km (human)/Km (mouse)], where Km factor = body weight (kg)/body surface area (m^2^). According to previous reports, the body surface areas of humans and mice are 1.62 and 0.007 m^2^, respectively, while body weights are 60 and 0.02 kg. Therefore, for a mouse weighing 0.02 kg, the mouse equivalent dose is approximately 0.05–2.9 mg/day. Finally, doses of 0.5, 1, and 2 mg/kg/day of PS‐NMPs were selected. Body weight was measured weekly, and the average daily water intake and feed consumption were recorded. All groups were intragastrically administered the dosage adjusted according to body weight once daily for five consecutive weeks, while the blank control group received normal saline. At the 5 weeks following exposure to the PS‐NMPs, the mice were fasted for 24 h and body weights were recorded prior to sampling. Under isoflurane anesthesia, the mice were euthanized by cervical dislocation. The entire colon was then dissected and collected. Colonic contents were aseptically collected using a sterile surgical blade and transferred into sterile 1.5 mL Eppendorf tubes, which were stored at −80°C for subsequent analysis.

### 2.4 Hematoxylin and Eosin Staining

Hematoxylin and eosin (H&E) staining was performed to analyze pathological changes to mouse colon tissue in accordance with the following procedure: Fixed tissues were rinsed with water and either directly dehydrated and embedded or stored long‐term in 75% ethanol at 4°C. Tissues retrieved from 75% ethanol were sequentially placed in 80%, 90%, and 100% ethanol for 1 h each, then incubated in 100% ethanol for 1 h. Subsequently, tissues were treated with a mixture of ethanol and xylene (1:1) for 30 min, followed by xylene I and xylene II, each for 30 min. Infiltration was then carried out in soft paraffin for 1 h and hard paraffin for 1 h. Molten pure paraffin was poured into an embedding mold placed on a cooling plate, and the tissue was positioned upright at the bottom using forceps. The paraffin‐embedded colon tissues were sliced using a microtome into 5‐μm thick sections, which were deparaffinized in xylene I for 10 min, followed by xylene II for 10 min. After removal of the xylene by washing twice in absolute ethanol for 1 min each, the sections were hydrated with a graded ethanol series (95% for 5 min, 90% for 5 min, and 85% for 5 min), then rinsed with tap water for 2 min, stained with hematoxylin for 15 min, and rinsed again with tap water for 1 min. Differentiation was achieved using 1% acid alcohol for 15 s until the sections turned pink, followed by rinsing with tap water for 1 min. Bluing was performed by soaking in tap water for 2 h. The sections were then stained with eosin for 1.5 min, rinsed three times with tap water, dehydrated with 85% ethanol for 5 min, 90% ethanol for 5 s, 95% ethanol I for 5 min, 95% ethanol II for 5 min, absolute ethanol I for 5 min, and absolute ethanol II for 5 min, cleared with xylene I for 10 min and xylene II for 10 min, and mounted on slides with neutral balsam. The mounted tissue sections were scanned using a high‐resolution panoramic imaging system and analyzed using ImageScope × 64 software (Leica Biosystems, Nussloch, Germany).

### 2.5 Alcian Blue‐Periodic Acid‐Schiff Staining

Tissue specimens were sliced into thin sections, which were fixed, dehydrated, and cleared, immersed in Alcian blue staining solution for 5 min, rinsed with tap water for 2 min, incubated in periodic acid solution for 15 min, washed twice with tap water and distilled water, stained with Schiff's reagent, typically for 15–30 min, and rinsed under running water for 5 min, stained with hematoxylin for 3–5 min, differentiated using a differentiation solution, rinsed with tap water, blued with a bluing solution, and finally washed under running water. Following sequential dehydration with absolute ethanol and absolute ethanol, the sections were cleared with xylene and mounted on slides with neutral balsam. Scanning was performed using a high‐resolution panoramic imaging system, and the number of goblet cells in the colonic mucosal epithelium was determined using ImageScope × 64 software.

### 2.6 Immunohistochemical Staining

Cells adhered to coverslips were washed three times for 3 min with phosphate‐buffered saline (PBS), fixed with 4% paraformaldehyde for 15 min, washed again three times for 3 min with PBS, permeabilized using 0.5% Triton X‐100 at room temperature for 20 min, and then washed three times for 3 min. Following removal of excess PBS using absorbent paper, the cells were blocked with normal goat serum at room temperature for 30 min. After removal of the blocking serum with absorbent paper without washing, the cells were incubated overnight at 4°C with a sufficient volume of diluted primary antibody in a humidified chamber. After washing three times for 3 min with PBS, excess liquid was removed with absorbent paper and the cells were incubated with the diluted secondary antibody in a humidified chamber at room temperature for 1 h and finally washed three times for 3 min with PBS. Then, the nuclei were stained with 4′,6‐diamidino‐2‐phenylindole in the dark for 5 min. After washing four times for 5 min with PBS, the cells were imaged using a high‐resolution panoramic scanning system.

### 2.7 Enzyme‐Linked Immunosorbent Assay

In accordance with the manufacturer's instructions, serum diamine oxidase (DAO) and D‐lactic acid (D‐LA) levels were measured using commercial enzyme‐linked immunosorbent Assay (ELISA) kits to determine changes to the intestinal permeability of mice. The expression levels of the inflammatory cytokines tumor necrosis factor‐alpha (TNF‐α), interleukin‐1 beta (IL‐1β), interleukin‐6 (IL‐6), and interleukin‐10 (IL‐10) in colonic tissues were detected using ELISA kits (Invitrogen Corporation, Carlsbad, CA, USA). The optical density of each sample was measured at a wavelength of 450 nm using a microplate reader. The expression levels of the inflammatory cytokines were calculated based on standard curves.

### 2.8 Quantitative Real‐Time PCR

The mRNA levels of colonic inflammatory cytokines (IL‐1β, IL‐6, IL‐10 and TNF‐α), intestinal barrier‐related proteins (claudin‐1, E‐cadherin, ZO‐1, occludin and MUC2), and pathway‐related proteins (TLR4, p‐FAK, NF‐κB and iNOS) were detected by quantitative real‐time PCR (qRT‐PCR) and graphs were generated. Colonic tissues (~30 mg) were meticulously minced in microcentrifuge tubes using ophthalmic scissors. Following the addition of 1 L TRIzol, the colonic tissues were lysed by pipetting and on ice for 5 min and then homogenized on ice three times using a homogenizer for 4 with 2‐s intervals. The homogenate was centrifuged at 12,000 × g for 10 min at 4°C, and the supernatant was transferred to a sterile microcentrifuge tube. The sample was allowed to stand for 5 min to ensure complete dissociation of nucleoprotein complexes. After adding chloroform (200 μL), the tube was inverted several times, incubated on ice for 5 min, and the sample was centrifuged at 12,000 × g for 15 min at 4°C. The upper aqueous phase was carefully transferred to a sterile microcentrifuge tube and thoroughly mixed with an equal volume of pre‐cooled isopropanol. The mixture was incubated on ice for 10 min and then centrifuged at 12,000 × g for 15 min at 4°C. Afterward, the supernatant was discarded, and the RNA pellet was washed with 1 mL of 75% ethanol by inverting the tube. Then, the sample was centrifuged at 10,000 × g for 5 min at 4°C. After discarding the supernatant, the washing step described above was repeated. Then, the tube was inverted and placed on clean absorbent paper to air‐dry at room temperature. The RNA pellet was dissolved in diethylpyrocarbonate‐treated water, mixed by pipetting, and the total RNA concentration was determined. RNA was stored at −80°C. Complementary DNA was synthesized from RNA using the PrimeScript RT reagent Kit (Perfect Real Time) (TaKaRa Bio Inc., Shiga) in accordance with the manufacturer's instructions. At least three independent replicates of all samples were assayed. Relative mRNA expression levels were calculated using the 2^‐ΔΔCT^ method and normalized to GAPDH expression. Relevant gene sequences were retrieved from the GenBankdatabase (https://www.ncbi.nlm.nih.gov/genbank/) for primer design and synthesis. The qRT‐PCR primer sequences are listed in Table 1.

**TABLE 1:** Primer sequences.

Gene	Sequences
Ocln	5′‐GGCAAGCGATCATACCCAGA‐3′
	5′‐GCTGCCTGAAGTCATCCACA‐3′
Tjp1	5′‐CTCACAGTACAGCCAGCCAG‐3′
	5′‐GGTGGGTCTGGTTTGGACAT‐3′
Muc2	5′‐TCTACCTCACCCACAAGCTG‐3′
	5′‐TGAGTGCATCTTCCCGGTTC‐3′
Ptk2	5′‐CTGCGGACCTTACTAGCCAC‐3′
	5′‐GCCTAAGTCGGAGTTCAGCA‐3′
Nfkb1	5′‐CGGAAGAGGGTCCACCAGAC‐3′
	5′‐AGCCTTCTCCCAAGAGTCGT‐3′
Nos2	5′‐ACTACTGCTGGTGGTGACAA‐3′
	5′‐GAAGGTGTGGTTGAGTTCTCTAAG‐3′
TLR4	5′‐TGGGTCAAGGAACAGAAGCA‐3′
	5′‐ATCCAACACTAAGGAGGTATTCATC‐3′
Il‐1β	5′‐AGACAACTGCACTACAGGCT‐3′
	5′‐TTGTCGTTGCTTGGTTCTCCTT‐3′
Il‐6	5′‐CCACTTCACAAGTCGGAGGCTTA‐3′
	5′‐TGCAAGTGCATCATCGTTGTTC‐3′
Il‐10	5′‐GCCAGAGCCACATGCTCCTA‐3′
	5′‐GATAAGGCTTGGCAACCCAAGTAA‐3′
Gapdh	5′‐GCTCTCATCATCGCCACAGA‐3′
	5′‐TTCGAGGTTCTGGGATGGGA‐3′

### 2.9 Cell Culture

FHC cells were cultured in F12 basal medium supplemented with 10% fetal bovine serum and 1% penicillin–streptomycin at 37°C under an atmosphere of 5% CO_2_/95% air.

### 2.10 Cell Viability

Cell viability was assessed using the Cell Counting Kit‐8 (CCK‐8) assay kit. ACell suspensions (100 μL) were added to the wells of 96‐well plates, which were precultured in an incubator for 24 h (37°C, 5% CO_2_). The culture medium was then replaced with medium containing appropriate concentrations of differently shaped PS‐NMPs. The plates were incubated for the desired durations. After incubation, CCK‐8 (10 μL) was added to each well, and the plates were further incubated for 1–4 h. Absorbance at 450 nm was measured using a microplate reader. Cell viability (%) was calculated as [A (treated) – A (blank)] / [A (0 treated) – A (blank)] × 100%. Suitable concentrations were selected for subsequent cell exposure experiments.

### 2.11 Small Interfering RNA

To provide a basis for subsequent validation of gene function in response to PS‐NMPs, knockdown of specific target genes (TLR4, FAK, and NF‐κB) in FHC cells was conducted via liposome‐mediated small interfering RNA (siRNA) transfection using Lipofectamine RNAiMAX transfection reagent (Thermo Fisher Scientific, Waltham, MA, USA). All siRNAs (against the target genes and negative control) were purified by high‐performance liquid chromatography to ensure a knockdown efficiency of ≥ 80%. The siRNA (1.5 μL, 20 μM) was diluted in 25 μL of Opti‐MEM serum‐free medium (Thermo Fisher Scientific). Separately, Lipofectamine RNAiMAX transfection reagent was diluted and incubated at room temperature for 15 min. The two solutions were then gently mixed by pipetting and incubated to form complexes. Following the addition of the siRNA–liposome complex (50 μL), each dish was incubated at 37°C for 12 h under an atmosphere of 5% CO_2_/95% air. The medium was subsequently replaced with complete medium containing 10% fetal bovine serum, and the cells were cultured for an additional 24 h.

### 2.12 Western Blots Analysis

Adherent cells in the logarithmic growth phase were seeded into 10 cm culture dishes at a density of 5 × 10^4^ cells per well and spread evenly. The cells were washed once with PBS, followed by the addition of 1–2 mL of cell lysis buffer. The mixture was allowed to stand on ice for 30 min to ensure complete lysis. The lysate was collected and centrifuged (12,000 rpm, 4°C, 20 min). The protein concentrations in 1‐μL aliquots of the supernatants from each tube were determined using the bicinchoninic acid (BCA) assay. In brief, BCA working solution was prepared as described in the manufacturer's instructions, was added to each well, and the plate was incubated in a water bath at a constant temperature of 37°C for 30 min. The optical density was measured at 562 nm using a full‐wavelength microplate reader, and the values were recorded. Based on the molecular weights of the target proteins, appropriate separating and stacking gels were selected. The proteins were separated by electrophoresis at a constant voltage of 150 V for 1 h and stopped when the bromophenol blue tracking dye reached the bottom edge of the separating gel. Then, the proteins were electroblotted onto polyvinylidene fluoride membranes, which were immersed in methanol for 1 min. The PVDF membranes were blocked with 10% skim milk powder on a shaker at room temperature for 1–2 h, washed three times for 10 min with buffer, and probed overnight on a shaker at 4°C with specific primary antibodies (Table 2). Following recovery of the primary antibody, the membranes were washed three times for 10 min with Tris‐buffered saline/Tween‐20, then incubated with an appropriate secondary antibody (dilution, 1:5000 in 10% Tris‐buffered saline/Tween‐20) at room temperature for 30 min. After recovery of the secondary antibody, the membranes were washed three times for 5 min with buffer.

**TABLE 2:** Antibodies and dilutions for western blot analysis.

Antibody	Dilution
Occludin	1:2000
E–cadherin	1:2000
ZO‐1	1:5000
MUC 2	1:2000
TLR 4	1:1000
FAK	1:2000
NF‐κB	1:5000
iNOS	1:200
GAPDH	1:10000
β‐actin	1:5000

### 2.13 Statistical Analysis

All statistical analyses were performed using IBM SPSS Statistics for Windows (version 27.0; IBM Corporation, Armonk, NY, USA). Differences among groups were compared by one‐way analysis of variance followed by Tukey's multiple comparison test. Data are presented as the mean ± standard deviation (SD). A probability (*p*) value ≤ 0.05 was considered statistically significant.

## 3 Results

### 3.1 Characterization of PS‐Fibers and PS‐Beads

The PS‐Fibers and PS‐Beads were characterized by SEM. As shown in Figure 1A–D and Table 3, the average length and average particle size of the PS‐Fibers were 10 μm and 500 nm, respectively. Meanwhile, the average diameter of the PS‐Beads was 500 nm.

**FIGURE 1:**
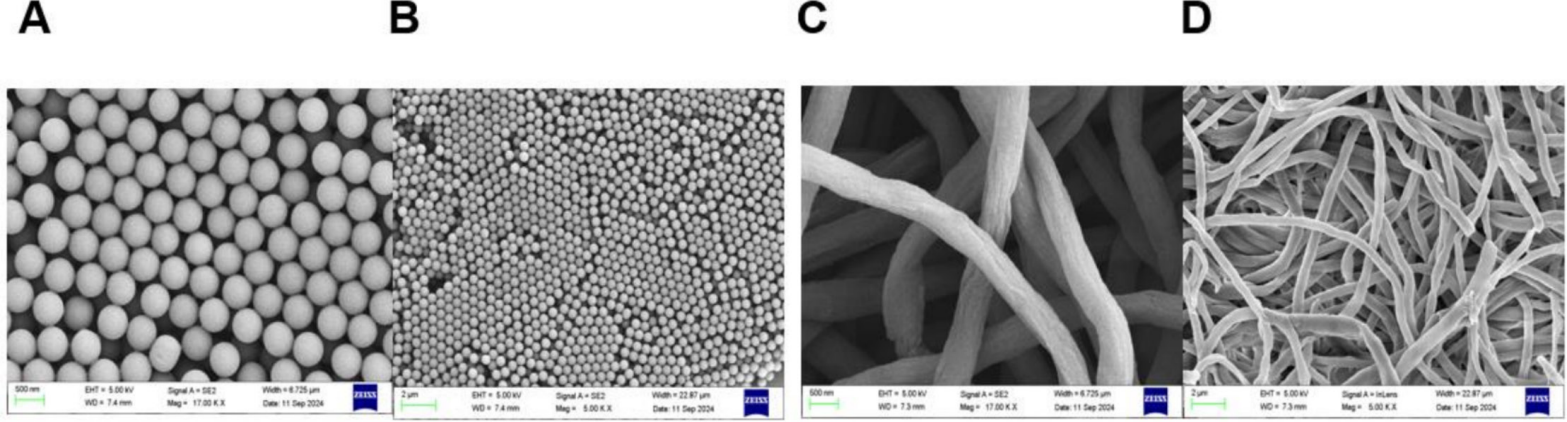
Representative SEM images of PS‐Beads (A, B) and PS‐Fibers (C, D).

**TABLE 3:** Physicochemical characterization data of PS‐Fibers and PS‐Beads after vortexing for 15 min.

Sample	Average length by SEM/μm	Average diameter by SEM/nm
PS‐Fibers	9.71 ± 1.58	632.75 ± 116.56
PS‐Beads	—	455.5 ± 3.42

### 3.2 Toxicity of PS‐Fibers and PS‐Beads on Mouse Colon Tissue

Oral exposure to PS‐Fibers and PS‐Beads alone for 5 weeks affected the colon length of mice. As compared to the negative control group, colon length was significantly reduced in the PS‐Fibers group (*p* < 0.01) and the PS‐Beads group, but this difference was not statistically significant (Figure 2A). Compared to the PS‐Beads group, colon length was significantly shorter in the PS‐Fibers group (*p* < 0.01). PS‐Fibers and PS‐Beads alone induced pathological damage to mouse colon tissue after 5 weeks of oral exposure. In the negative control group, the mucosal structure of the colon tissue was normal, with no obvious pathological changes. Compared to the negative control group, the low‐dose PS‐Fibers group exhibited mild inflammatory cell infiltration (Figure 2B), while the medium‐dose PS‐Fibers group showed marked inflammatory cell infiltration, thinning of the mucosal layer, disordered crypt architecture, and an increased number of goblet cells, whereas the high‐dose PS‐Fibers group displayed significant inflammatory cell infiltration accompanied by inflammatory exudation, disappearance of the mucosal layer, disordered crypt architecture, and a pronounced increase in goblet cells. Compared to the negative control group, the low‐dose PS‐Beads group showed mild inflammatory cell infiltration, the medium‐dose PS‐Beads group exhibited obvious inflammatory cell infiltration, and the high‐dose PS‐Beads group demonstrated marked inflammatory cell infiltration with inflammatory exudation. Compared to the low‐dose PS‐Beads group, more goblet cells were observed in the low‐dose PS‐Fibers group. Relative to the medium‐dose PS‐Beads group, the medium‐dose PS‐Fibers group exhibited thinning of the mucosal layer, disordered crypt architecture, and an increased number of goblet cells. In comparison with the high‐dose PS‐Beads group, the high‐dose PS‐Fibers group showed disappearance of the mucosal layer, disordered crypt architecture, and a significant increase in the number of goblet cells. The colon exhibited different sensitivities to PS‐NMPs of different shapes. Exposure to PS‐Beads induced varying degrees of inflammatory injury in mouse colon tissue, while exposure to PS‐Fibers resulted in inflammatory injury, thinning of the mucosal layer, disordered crypt architecture, and a compensatory increase in the number of goblet cells. Compared to PS‐Beads, PS‐Fibers exerted greater toxic effects on mouse colon tissue.

**FIGURE 2:**
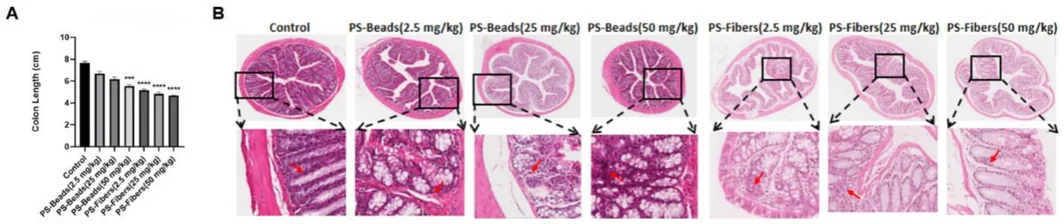
Toxicity of PS‐Fibers and PS‐Beads on mouse colon tissue. (A) Effects of PS‐Fibers and PS‐Beads on mouse colon length. Data are presented as the mean ± SD (*n* = 3). *p < 0.05, **p < 0.01, ***p < 0.001 versus negative control group. (B) Representative images of H&E‐stained colon tissues from mice in each group. Red arrows indicate inflammatory cells. Scale bars: upper panel, 50 μm; lower panel, 20 μm; *n* = 3.

### 3.3 Effects of PS‐Fibers and PS‐Beads on the Colonic Physical Barrier

Changes to the intestinal permeability of the mouse colon induced by exposure to PS‐Fibers and PS‐Beads were assessed by measuring serum levels of DAO and D‐LA using ELISA kits. As shown in Figure 3A,B, both shapes of PS‐NMPs significantly increased intestinal permeability, with a more pronounced effect observed in the PS‐Fibers group. Compared to the negative control group, serum levels of both DAO and D‐LA were significantly elevated in all groups exposed to PS‐Fibers (*p* < 0.01). In the low‐dose PS‐Beads group, D‐LA levels were slightly increased (*p* < 0.05), while there were no significant differences in DAO levels (*p* > 0.05). In the medium‐ and high‐dose PS‐Beads groups, both D‐LA and DAO levels were significantly elevated (*p* < 0.01). At the same concentration, DAO and D‐LA levels were significantly higher in the PS‐Fibers group than the PS‐Beads group. Compared to the negative control group, mRNA expression levels of occludin and ZO‐1 were reduced in the PS‐Fibers and PS‐Beads groups (Figure 3C,D). Significant reductions in occludin and ZO‐1 mRNA expression (*p* < 0.01) were observed in the high‐dose PS‐Beads group and all PS‐Fibers groups (low, medium, and high). Immunohistochemical staining and western blot analysis were performed to compare the expression levels and distributions of the tight junction proteins ZO‐1 and occludin in the colonic mucosal epithelium (Figure 3E–J). Occludin was primarily localized at the margins and lateral surfaces of colonic epithelial cells, specifically at the intercellular junctions. Compared to the negative control group, occludin expression was lower in the colonic epithelium of the PS‐Fibers and PS‐Beads groups and exhibited a uniform but discontinuous distribution. Furthermore, occludin expression was significantly lower in the PS‐Fibers group than the PS‐Beads group (*p* < 0.01). Meanwhile, ZO‐1 was regularly distributed along the plasma membranes among the colonic epithelial cells in all groups. Relative to the negative control group, ZO‐1 expression was markedly reduced (*p* < 0.01) in the PS‐Fibers and PS‐Beads groups, showing an irregular and discontinuous distribution pattern, which was consistent with the expression profile of occludin.

**FIGURE 3:**
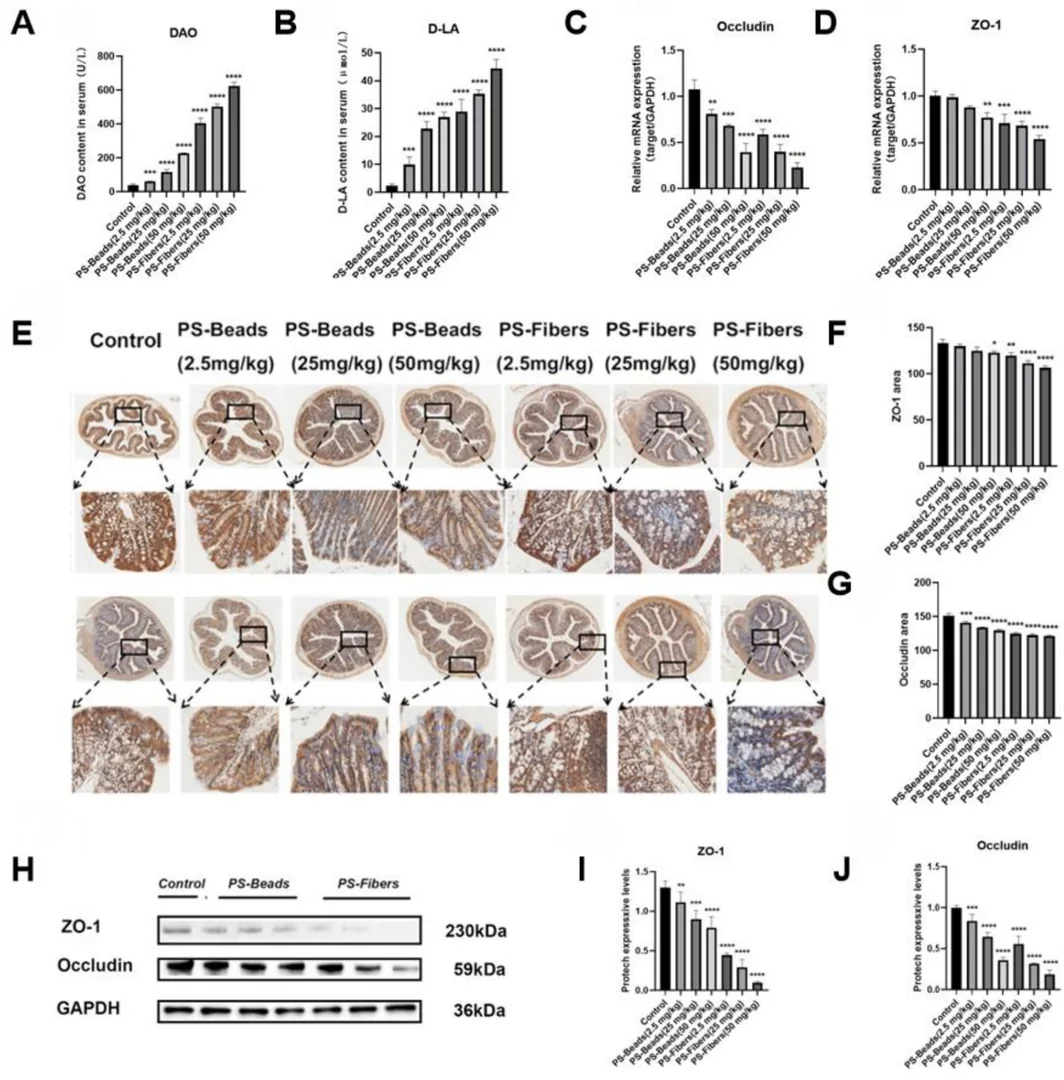
Effects of PS‐Fibers and PS‐Beads on the colonic physical barrier. (A) Effects of PS‐Fibers and PS‐Beads on the content of DAO in mouse colon tissues. (B) Effects of PS‐Fibers and PS‐Beads on the content of D‐LA in mouse colon tissues. Data are presented as the mean ± SD (*n* = 3). (C and D) Occludin and ZO‐1 mRNA expression levels of mouse colon tissue. (E) Immunohistochemical staining of ZO‐1 and occludin. Red arrows indicate the localization of ZO‐1 and occludin (scale bars: upper panel, 50 μm; lower panel, 20 μm). (F and G) Quantitative immunohistochemical analysis of occludin and ZO‐1 in mouse colon tissues. (H–J) Protein expression levels of the tight junction proteins ZO‐1 and occludin in mouse colon tissues after exposure to PS‐Fibers or PS‐Beads. Quantitative analysis of occludin and ZO‐1 protein band intensities in mouse colon tissues. *p < 0.05, **p < 0.01, ***p < 0.001 versus negative control group.

### 3.4 Effects of PS‐Fibers and PS‐Beads on the Colonic Chemical Barrier

The qRT‐PCR results (Figure 4A) demonstrated that, compared to the negative control group, MUC2 and E‐cadherin mRNA expression levels were significantly decreased in all PS‐Fibers groups (low, medium, and high) (*p* < 0.01). In the low‐dose PS‐Beads group, there was no significant change to MUC2 mRNA expression, while E‐cadherin mRNA expression was significantly reduced (*p* < 0.01). The medium‐dose PS‐Beads group exhibited a significant decrease in MUC2 mRNA expression (*p* < 0.05) and a highly significant decrease in E‐cadherin mRNA expression (*p* < 0.01). In the high‐dose PS‐Beads group, the mRNA expression levels of both MUC2 and E‐cadherin were significantly lower (*p* < 0.01). The expression and distribution of colonic MUC2 were detected and observed by immunohistochemical staining. As shown in Figure 4B, MUC2 was primarily located within the intestinal mucus layer. As compared to the negative control group, the PS‐Fiber and PS‐Beads groups exhibited a discontinuous and uneven distribution of MUC2 protein on the colonic epithelial surface, with significantly decreased expression levels (*p* < 0.01). The Alcian blue–periodic acid‐Schiff (AB‐PAS) staining results (Figure 4C,D) revealed that in the negative control group, goblet cells were densely distributed on both sides of the crypts, exhibiting a plump, rounded morphology and abundant mucus secretion. As compared to the negative control group, the number of goblet cells was significantly reduced (*p* < 0.01) with a relatively sparse distribution in the PS‐Fiber and PS‐Beads groups. Furthermore, the number of goblet cells was significantly lower in the PS‐Fiber groups than the PS‐Beads groups (*p* < 0.01), indicating that exposure to both PS‐Fibers and PS‐Beads led to reduced numbers of intestinal goblet cells. Western blot and qRT‐PCR analyses were performed to detect MUC2 and E‐cadherin protein levels in mouse colon tissue (Figure 4E–I). Relative to the negative control group, the protein and mRNA expression levels of both MUC2 and E‐cadherin were significantly reduced in all PS‐Fiber groups (low, medium, and high) (*p* < 0.01). In the low‐ and medium‐dose PS‐Beads groups, there were no significant changes to MUC2 and E‐cadherin protein levels. However, MUC2 protein expression was significantly decreased in the high‐dose PS‐Beads group (*p* < 0.05), whereas E‐cadherin protein expression was highly significantly decreased (*p* < 0.01). In summary, PS‐Fibers and PS‐Beads induced colonic chemical barrier dysfunction.

**FIGURE 4:**
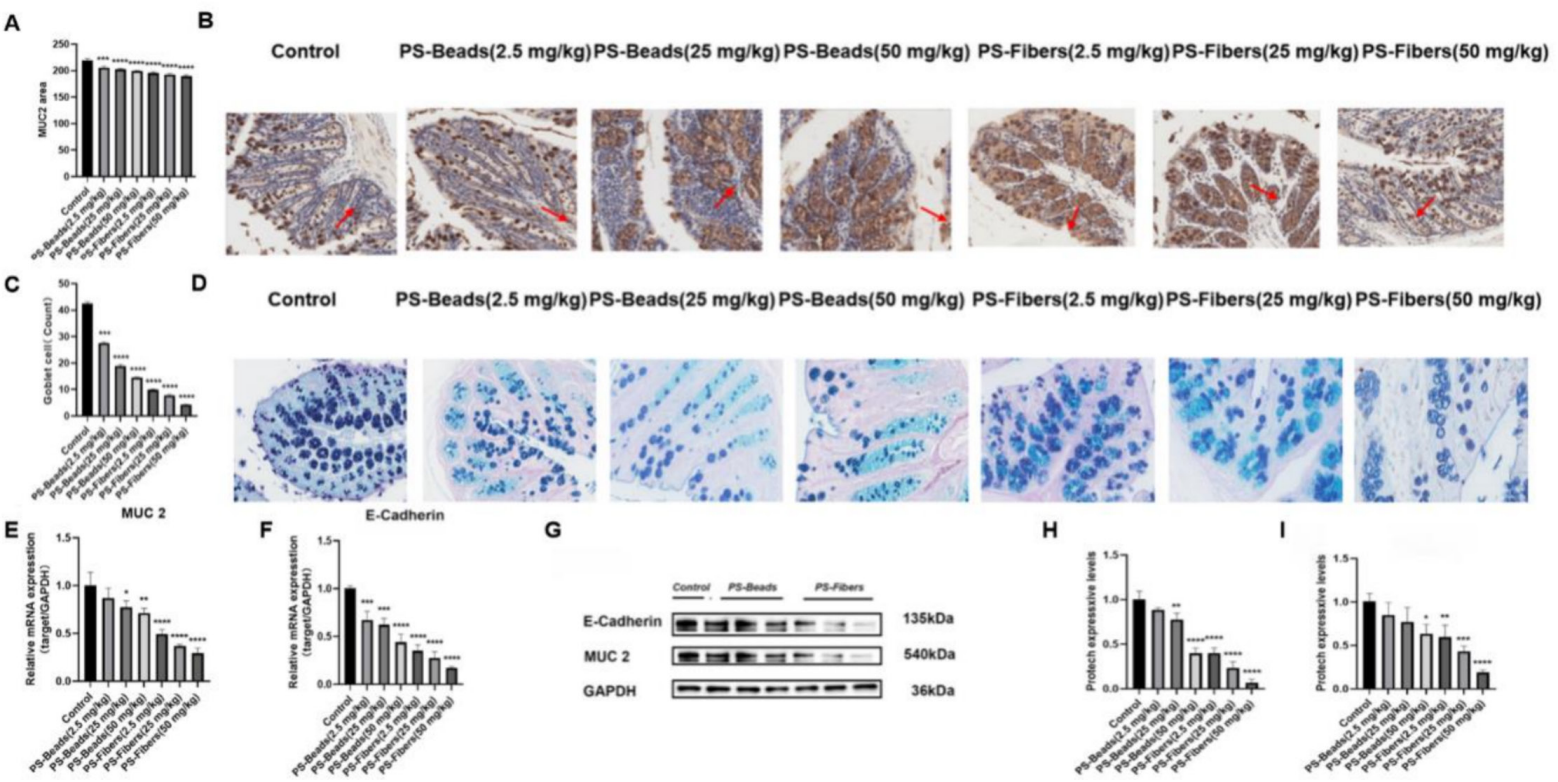
Effects of PS‐Fibers and PS‐Beads on the colonic chemical barrier. (A and B) Positive immunohistochemical staining for MUC2 in mouse colon tissue. The red arrow indicates the location of MUC2 (scale bar: 50 μm). Quantitative immunohistochemical analysis of MUC2 in mouse colon tissue. (C and D) AB‐PAS staining of colonic tissue (scale bar: 20 μm). Quantitative analysis of goblet cell numbers based on AB‐PAS staining of colonic tissue. (E and F) MUC2 and E‐cadherin mRNA expression levels in mouse colon tissue. (G) Representative western blots of MUC2 and E‐cadherin proteins in mouse colon tissue. (H and I) Relative protein expression levels of MUC2 and E‐cadherin in mouse colon tissue. *p < 0.05, **p < 0.01, ***p < 0.001 versus negative control group.

### 3.5 Effects of PS‐Fibers and PS‐Beads on the Colonic Immune Barrier

The qRT‐PCR results (Figure 5A–D) demonstrated that, compared to the negative control group, the mRNA expression levels of IL‐1β, IL‐6, and TNF‐α were significantly increased (*p* < 0.01), while the mRNA expression level of IL‐10 was significantly decreased (*p* < 0.01) in all PS‐Fibers groups (low, medium, and high). In the low‐ and medium‐dose PS‐Beads groups, no significant changes were observed in the mRNA expression levels of the immune factors IL‐1β, IL‐6, and TNF‐α, although mRNA expression level of IL‐10 was significantly reduced (*p* < 0.01). In the high‐dose PS‐Beads exposure group, no significant changes were found in the mRNA expression levels of IL‐1β and TNF‐α, whereas mRNA expression of IL‐6 was significantly elevated (*p* < 0.01), while that of IL‐10 was significantly decreased (*p* < 0.01). The ELISA results (Figure 5E–H) indicated that, relative to the negative control group, the mRNA expression levels of the pro‐inflammatory cytokines IL‐1β, IL‐6, and TNF‐α were significantly reduced (*p* < 0.01), while the mRNA expression level of the anti‐inflammatory cytokine IL‐10 was significantly decreased (*p* < 0.01) in all PS‐Fibers groups (low, medium, and high). Similarly, in all PS‐Beads groups (low, medium, and high), the mRNA expression levels of the pro‐inflammatory cytokines IL‐1β, IL‐6, and TNF‐α, as well as the anti‐inflammatory cytokine IL‐10, were significantly reduced (*p* < 0.01). In summary, these results indicate that both PS‐Fibers and PS‐Beads can affect the colonic immune barrier, with PS‐Fibers exerting a more pronounced impact.

**FIGURE 5:**
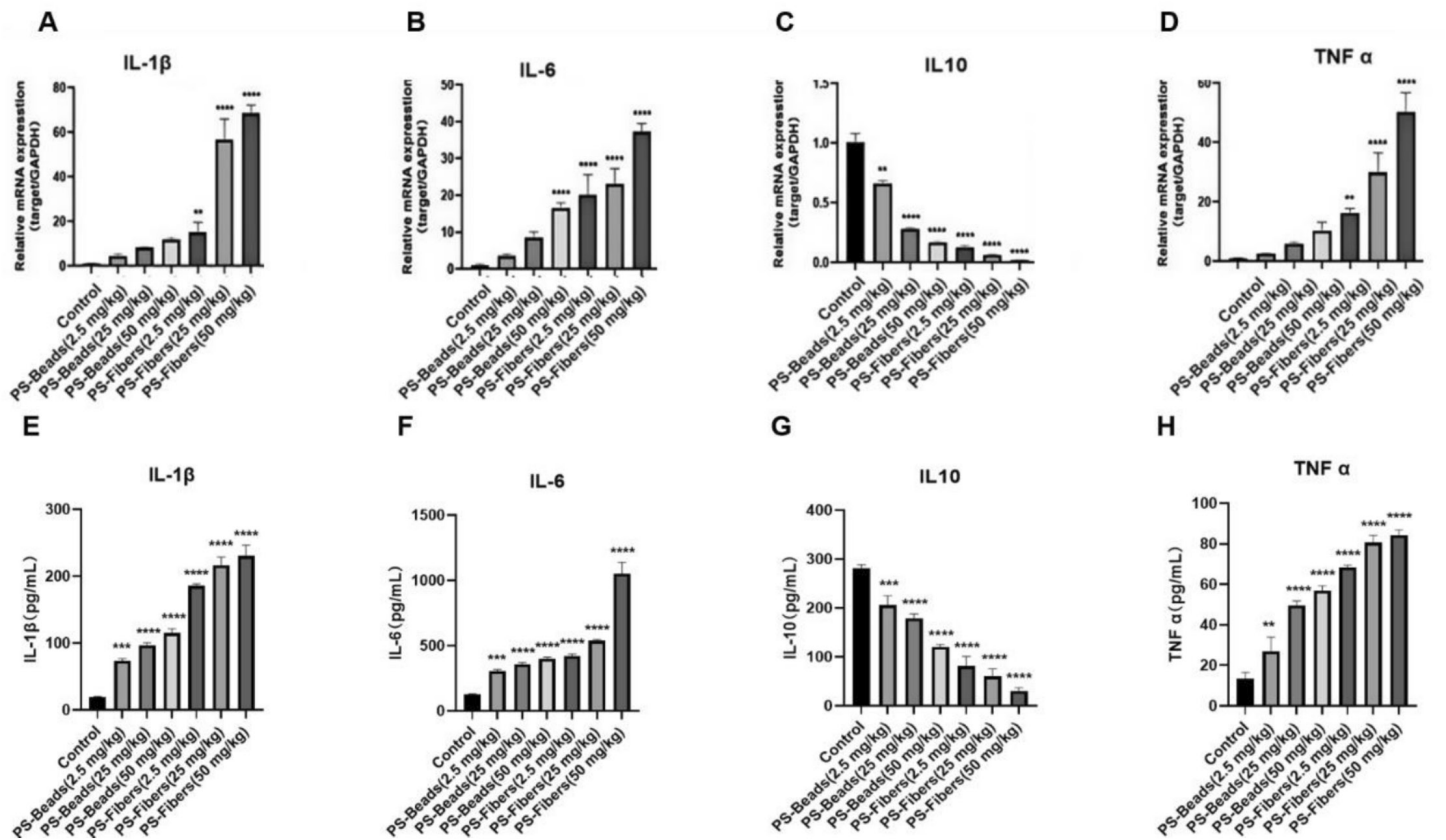
Effects of PS‐Fibers and PS‐Beads on the colonic immune barrier. (A–D) Relative mRNA expression levels of IL‐1β, IL‐6, IL‐10, and TNF‐α in colon tissue. (E–H) The mRNA expression levels of the inflammatory cytokines IL‐1β, IL‐6, IL‐10, and TNF‐α in mouse colon tissue. *p < 0.05, **p < 0.01, ***p < 0.001 versus negative control group.

### 3.6 Effects of PS‐Fibers and PS‐Beads on Expression of TLR4, NF‐κB, and iNOS in Mouse Colon Tissue

The qRT‐PCR results (Figure 6A–C) demonstrated that, compared to the negative control group, the mRNA expression levels of TLR4, NF‐κB, and iNOS were significantly increased (*p* < 0.05) in all PS‐Fibers‐exposed groups (low, medium, and high), exhibiting a dose‐dependent manner. In the low‐dose PS‐Beads group, the mRNA expression levels of TLR4 and iNOS showed no significant changes (*p* > 0.05). However, the mRNA expression levels of TLR4, NF‐κB, and iNOS were significantly elevated (*p* < 0.01) in the medium‐ and high‐dose PS‐Beads groups. Compared to the PS‐Beads groups, iNOS mRNA expression was significantly higher in the PS‐Fibers groups, whereas no significant differences were observed in TLR4 and NF‐κB mRNA expression levels. Western blot analysis was employed to detect the expression and distribution patterns of pathway‐related proteins (TLR4, p‐FAK, NF‐κB and iNOS) in mouse colon tissue, as shown in Figure 6D–G. Relative to the negative control group, the protein expression levels of TLR4, NF‐κB, and iNOS were significantly increased (*p* < 0.01) in the colon tissue of the PS‐Fiber group. In the low‐dose PS‐Beads group, the protein levels of TLR4 and NF‐κB were markedly elevated (*p* < 0.05), while the iNOS protein level was significantly increased (*p* < 0.01). The protein expression levels of TLR4, NF‐κB, and iNOS were all significantly increased (*p* < 0.01) in the medium‐ and high‐dose PS‐Beads groups, showing a dose–response relationship. Compared to the PS‐Beads groups, iNOS protein expression was significantly higher in the PS‐Fibers group (*p* < 0.01), whereas there were no significant changes in TLR4 and NF‐κB protein levels.

**FIGURE 6:**
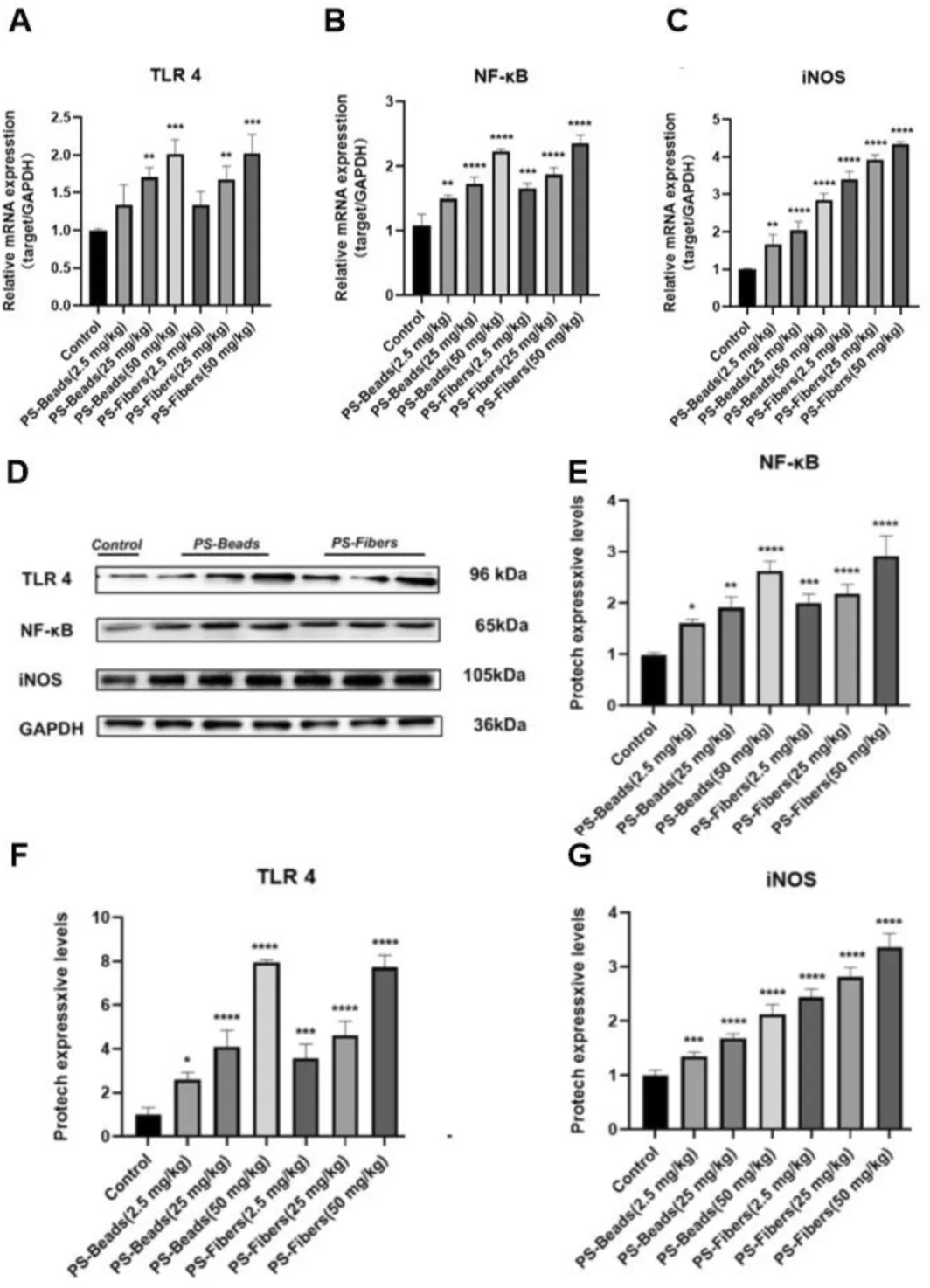
PS‐Fibers and PS‐Beads effected the expression of TLR4, NF‐κB, and iNOS in mice colon tissue. (A–C) The mRNA expression level of TLR4, NF‐κb, and iNOS in the colon. (D‐G) Representative Western blot images and quantitative analysis of the pathway‐related proteins TLR4, NF‐κB, and iNOS in colon tissue. Compared with the control group, *p < 0.05, **p < 0.01, ***p < 0.001.

### 3.7 PS‐Fibers and PS‐Beads Induced Colonic Intestinal Barrier Injury via the TLR4/NF‐κB/iNOS Pathway

The effects of PS‐Fibers and PS‐Beads in colonic intestinal barrier injury via the TLR4/NF‐κB/iNOS pathway are shown in Figure 7A–D. As compared to the negative control group, the protein expression levels of TLR4, NF‐κB, and iNOS were significantly increased in the PS‐Fibers group (all, *p* < 0.05), indicating sustained activation of the TLR4/NF‐κB pro‐inflammatory pathway. Compared to the PS‐Fibers group, TLR4 expression was markedly reduced in the PS‐Fibers + siTLR4 group (*p* < 0.01), and the protein levels of NF‐κB and iNOS were significantly decreased (*p* < 0.01). In comparison with the PS‐Fibers group, no significant difference in TLR4 protein expression was observed between the PS‐Fibers + siNF‐κB group and the negative control group, while the protein levels of NF‐κB and iNOS were significantly reduced (*p* < 0.01). As shown in Figure 7E–I, the intestinal barrier function proteins (ZO‐1, occludin and E‐cadherin) were expressed at basal levels in the negative control group, and MUC2 was maintained at a steady state. Compared to the negative control group, protein expression of ZO‐1 was decreased (*p* < 0.01), occludin protein expression was downregulated (*p* < 0.01), E‐cadherin protein expression was significantly reduced (*p* < 0.01), and MUC2 levels were markedly lowered (*p* < 0.01) in the PS‐Fibers group, suggesting that PS‐Fibers directly damage the intestinal barrier structure. Relative to the PS‐Fibers group, the expression levels of the intestinal barrier proteins ZO‐1, occludin, E‐cadherin, and MUC2 were significantly restored in the PS‐Fibers + siTLR4 group (*p* < 0.01), confirming that PS‐Fibers induce colonic intestinal barrier injury and indicating the critical role of TLR4 in this process. Compared to the PS‐Fibers group, the expression levels of the intestinal barrier proteins ZO‐1, occludin, E‐cadherin, and MUC2 were recovered to varying degrees in the PS‐MPs‐Fibers + siNF‐κB group (all, *p* < 0.01), suggesting the key role of NF‐κB in intestinal barrier injury induced by PS‐Fibers.

**FIGURE 7:**
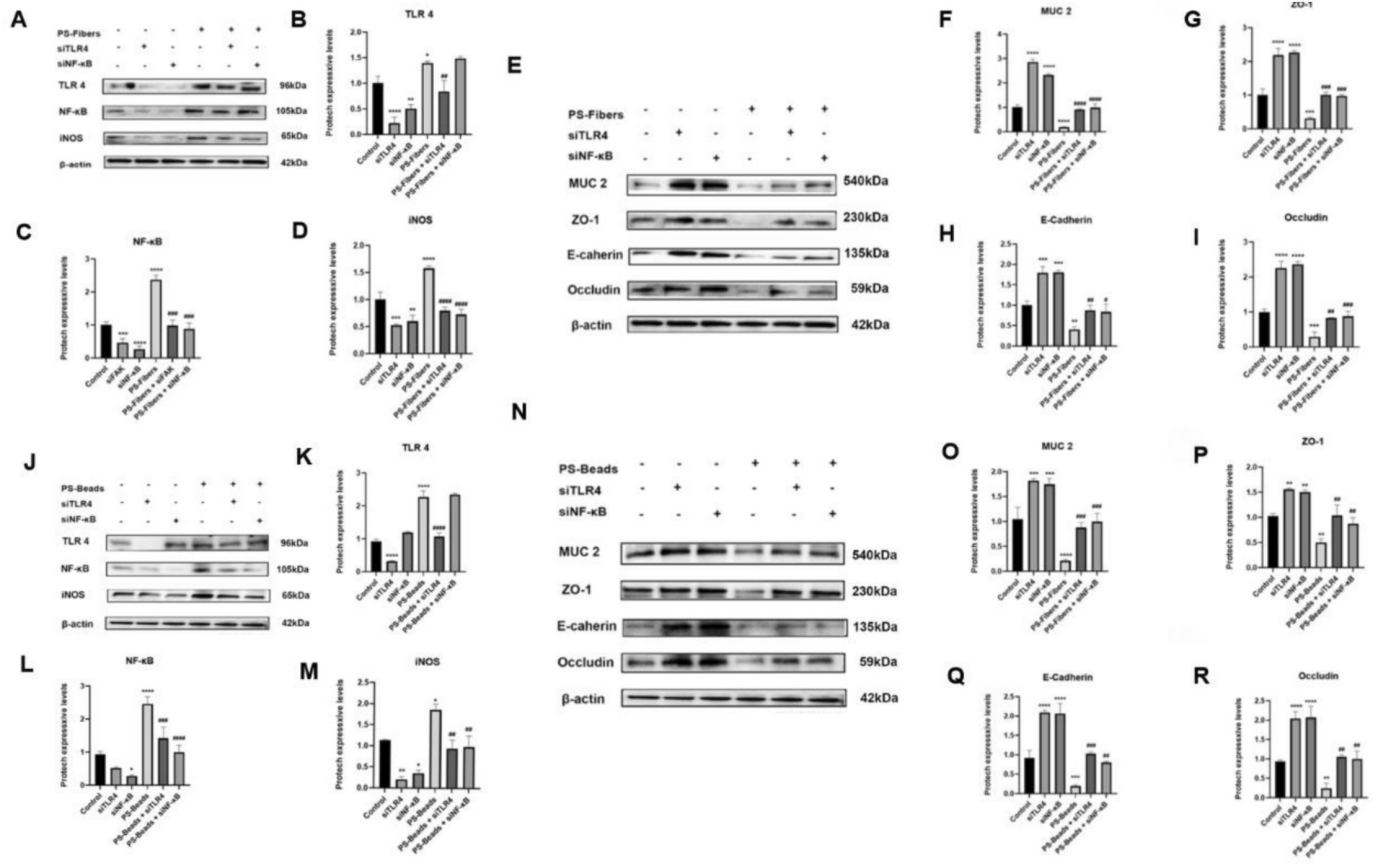
PS‐Fibers and PS‐Beads induced colonic intestinal barrier injury via the TLR4/NF‐κB/iNOS pathway. (A–D) Expression levels of proteins related to the TLR4/NF‐κB/iNOS pathway after treatment with PS‐Fibers and siTLR4 or siNF‐κB. (E–I) Expression levels of proteins related to the MUC2, ZO‐1, E‐cadherin, and occludin pathway after treatment with PS‐Fibers and siTLR4 or siNF‐κB. (J–M) Expression levels of proteins related to the TLR4/NF‐κB/iNOS pathway after treatment with PS‐Beads and siTLR4 or siNF‐κB. (N–R) Expression levels of proteins related to the MUC2, ZO‐1, E‐cadherin, and occludin pathway after treatment with PS‐Beads and siTLR4 or siNF‐κB. *p < 0.05, **p < 0.01 versus negative control group; ^#^ *p* < 0.05, ^##^ *p* < 0.01 versus PS‐Fibers and PS‐Beads groups; *n* = 3.

As shown in Figure 7J–M, relative to the negative control group, the protein expression levels of TLR4, NF‐κB, and iNOS were significantly elevated in the PS‐Beads group (*p* < 0.05), indicating sustained activation of the TLR4/NF‐κB pro‐inflammatory pathway. In the PS‐Beads + siTLR4 group, TLR4 expression was significantly reduced compared to the PS‐Beads group (*p* < 0.01), and the protein levels of NF‐κB and iNOS were also markedly decreased (*p* < 0.01). In the PS‐Beads + siNF‐κB group, there was no significant difference in TLR4 expression relative to the negative control group, while NF‐κB and iNOS protein levels were significantly lower (*p* < 0.01) than in the PS‐Beads group. As shown in Figure 7N–R, the intestinal barrier function proteins ZO‐1, occludin, and E‐cadherin were expressed at basal levels in the negative control group, and MUC2 was maintained in a homeostatic state. Compared to the negative control group, the PS‐Beads group showed decreased ZO‐1 expression (*p* < 0.01), down‐regulated occludin protein levels (*p* < 0.01), significantly reduced E‐cadherin expression (*p* < 0.01), and markedly lowered MUC2 levels (*p* < 0.01), indicating that PS‐Beads directly damage the intestinal barrier structure. Relative to the PS‐Beads group, the PS‐Beads + siTLR4 group exhibited significant restoration of ZO‐1, occludin, E‐cadherin, and MUC2 protein expression (*p* < 0.01), confirming that PS‐Beads induced colonic intestinal barrier damage. In the PS‐Beads + siNF‐κB group, the expression levels of ZO‐1, Occludin, E‐cadherin, and MUC2 were all restored to varying degrees (all, *p* < 0.01) compared to the PS‐Beads group. In summary, both PS‐Fibers and PS‐Beads induced colonic intestinal barrier damage via the TLR4/NF‐κB/iNOS axis.

### 3.8 PS‐Fibers Induced Colonic Intestinal Barrier Injury via the FAK/NF‐κB/iNOS Pathway in Contrast to PS‐Beads

As a nonreceptor tyrosine kinase, FAK plays a critical role in cell adhesion, migration, and mechanotransduction. Upon activation, FAK can also influence NF‐κB expression. Therefore, we examined the effects of PS‐Fibers and PS‐Beads on p‐FAK in mouse colon. Compared to the negative control group, the protein expression level of FAK was significantly increased in the PS‐Fibers group (*p* < 0.01) in a dose‐dependent manner. In contrast, no significant changes were observed in either FAK mRNA or protein expression levels in the PS‐Beads group (Figure 8A–C). Thus, we hypothesized that PS‐Fibers may also induce colonic inflammation via activation of p‐FAK and subsequent influence of NF‐κB and iNOS. To test this hypothesis, FHC cells were co‐treated with PS‐Fibers and siFAK or siNF‐κB. As shown in Figure 8D–G, compared to the negative control group, the protein expression levels of p‐FAK, NF‐κB, and iNOS were significantly elevated in the PS‐Fibers group (all, *p* < 0.01), indicating sustained activation of the FAK/NF‐κB pro‐inflammatory pathway. In the PS‐Fibers + siFAK group, p‐FAK expression was significantly reduced (*p* < 0.01) and the protein levels of NF‐κB and iNOS were markedly decreased (*p* < 0.01) compared to the PS‐Fibers group. In the PS‐Fibers + siNF‐κB group, p‐FAK protein expression showed no significant difference from the negative control group, while NF‐κB and iNOS protein levels were significantly reduced (*p* < 0.01) compared to the PS‐Fibers group (Figure 8H,I). In summary, while PS‐Beads induced NF‐κB and iNOS only through TLR4, PS‐Fibers up‐regulated the expression of NF‐κB and iNOS via both p‐FAK and TLR4. This dual regulatory effect explains why PS‐Fibers caused more severe colonic intestinal injury than PS‐Beads.

**FIGURE 8:**
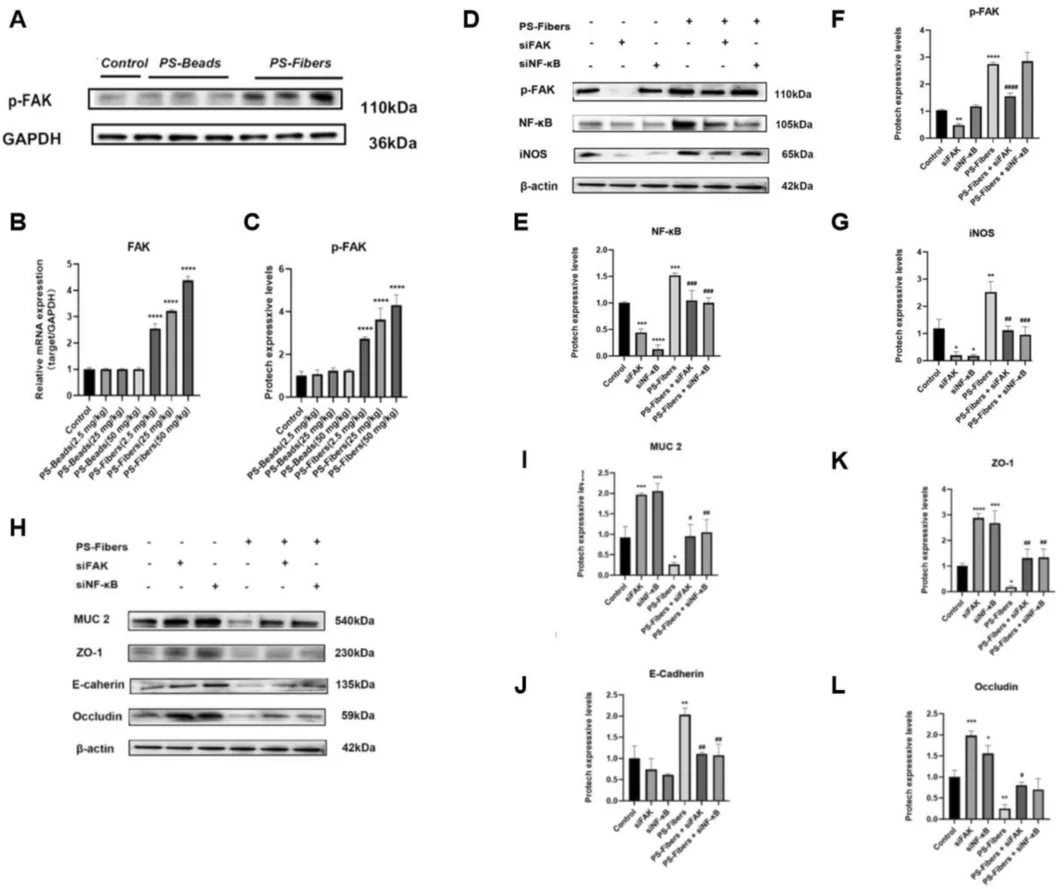
PS‐Fibers induced colonic intestinal barrier injury via the FAK/NF‐κB/iNOS pathway. (A and B) Representative western blot images and quantitative analysis of FAK in mouse colon tissue. (C) FAK mRNA expression levels in mouse colon tissue. (D–J) Expression levels of proteins associated with the FAK/NF‐κB/iNOS pathway. (H–L) Expression levels of proteins associated with colonic barrier injury (MUC2, ZO‐1, E‐cadherin, and occludin). *p < 0.05, **p < 0.01 versus negative control group; ^#^ *p* < 0.05, ^##^ *p* < 0.01 versus PS‐Fibers group; *n* = 3.

## 4 Discussion

As a class of emerging environmental pollutants, the impact of morphological heterogeneity of NMPs on potential toxicological effects has become a research focus in the field of environmental toxicology. This study systematically investigated the differential mechanisms of fibrous and microsphere forms of PS‐NMPs in colonic barrier damage both in vivo and in vitro. The toxicity of the two shapes of PS‐NMPs differed, with PS‐Fibers exhibiting greater toxicity than PS‐Beads. Both shapes induced toxic effects via activation of the NF‐κB/iNOS pathway, but significant differences were observed in their upstream molecular mechanisms, as PS‐Beads acted through the TLR4/NF‐κB/iNOS pathway, while PS‐Fibers additionally involved the FAK/NF‐κB/iNOS pathway. These findings provide new experimental evidence to elucidate the morphology‐dependent intestinal toxicity mechanisms of NMPs and establish a foundation to assess the health risks posed by different morphological forms in the environment.

The morphological diversity of environmental NMPs (e.g., fibers, films and fragments) contributes to the complexity of their toxic effects. In this study, characterization by SEM revealed that PS‐Beads exhibited a uniform particle size (500 nm), whereas PS‐Fibers displayed a characteristic high aspect ratio (length, 10 μm, diameter, 500 nm). This morphological difference may be a key factor contributing to the divergence in the toxicological effects between PS‐Fibers and PS‐Beads. Animal experiments demonstrated that both types of PS‐NMPs induced shortening of the colon, damage to the mucosal layer, increased intestinal permeability (elevated levels of DAO and D‐LA), and infiltration of inflammatory cells in mice. However, at the same dosage, PS‐Fibers caused more severe pathological damage. These findings are consistent with the results of an in vivo study conducted by Saenen et al. that PS‐NMPs can induce colonic toxicity (primarily manifested pathologically) (Saenen et al. 2023) and that fibrous PS‐NMPs (8.9 ± 10.1 μm) induced significantly stronger oxidative stress and mitochondrial dysfunction in human colorectal adenocarcinoma cells (Caco‐2) than spherical particles of the same size, suggesting that particle morphology may modulate toxicity by influencing particle–cell interactions (e.g., adhesion and endocytosis efficiency). Furthermore, a study by Chen et al. found a dose‐dependent toxicological (Chen et al. 2025). In their 60‐day mouse gavage model, a positive correlation was observed between the dose of NMPs and reduced colonic mucus secretion, as well as increased intestinal permeability.

The integrity of the intestinal barrier (physical, chemical, and immune) is essential to maintain intestinal homeostasis. This study revealed the damaging effects of PS‐NMPs on the colonic barrier at multiple levels. Regarding the physical barrier, the expression levels of the tight junction proteins occludin and ZO‐1 along with the epithelial adhesion molecule E‐cadherin were significantly decreased following exposure to PS‐NMPs, with a more pronounced reduction in the PS‐Fiber group. This finding is consistent with disruption of the intestinal mucosal physical barrier induced by NMPs reported in a mouse gavage model (Liang et al. 2021). Through further mechanistic studies, Yang et al. found that expression of tight junction proteins is regulated by the NF‐κB/iNOS pathway (Yang et al. 2025). Activation of NF‐κB/iNOS downregulates expression of ZO‐1 and occludin via inhibition of their transcriptional promoters. This mechanism was validated in this study's siRNA interference experiments, which showed that knockdown of NF‐κB significantly alleviated the decreased expression of tight junction proteins induced by both shapes of PS‐NMPs. Apart from PS‐NMPs, polyvinyl chloride (PVC)‐NMPs with a diameter of 2 μm also caused colonic barrier dysfunction and the decrease of MUC2 (Chen et al. 2022). Furthermore, polyethylene (PE)‐NMPs with a diameter of 5 μm induce colonic intestinal barrier damage; the levels of ZO‐1 and claudin‐1 were significantly decreased in PE‐NMPs groups (El Gazzar et al. 2023). These findings suggest that diverse types of NMPs can induce intestinal barrier dysfunction. Future studies will systematically compare the relative toxic potency of different NMPs types to delineate their differential pathogenic effects and underlying mechanisms.

In the context of the chemical barrier, the number of goblet cells and the expression level of MUC2 are key indicators of intestinal mucus layer function. In this study, AB‐PAS staining revealed that exposure to PS‐NMPs led to a reduction in the number of goblet cells, with a more pronounced decrease observed in the PS‐Fibers group. Concurrently, both the mRNA and protein expression levels of MUC2 were significantly reduced. As the first line of chemical defense in the intestine, the mucus layer prevents pathogens and foreign particles from adhering to epithelial cells. Decreased expression of MUC2 compromises this protective function, thereby increasing the risk of intestinal infections. This finding is consistent with a study by Zangene et al. with a mouse gavage model, where exposure to PS‐NMPs was shown to inhibit intestinal mucus secretion and disrupt mucin secretion, with mucus layer thickness negatively correlated with particle concentration (Zangene et al. 2025). Notably, the reduction in MUC2 expression induced by PS‐Fibers in this study (~55%) was significantly greater than in the PS‐Beads group (~30%), which may be attributed to the tendency of PS‐Fibers to adhere to and disrupt the mucus layer structure more readily. It is hypothesized that fibrous shapes, due to the elongated axis, can become embedded in the mucus layer, leading to a looser colonic physical structure and consequently greater toxicity.

Regarding the immune barrier, exposure to PS‐NMPs significantly up‐regulated the expression levels of the pro‐inflammatory cytokines IL‐6, IL‐1β, and TNF‐α, while down‐regulating expression of the anti‐inflammatory cytokine IL‐10. Moreover, the inflammatory imbalance was more severe in the PS‐Fibers group. These results are consistent with the findings of Zhai et al. with an acute mouse exposure model, where mice fed PS‐NMPs exhibited reduced MUC2 expression and elevated levels of inflammatory cytokines (TNF‐α, IL‐6, IL‐1β, and IL‐17) (Zhai et al. 2024). Li et al. reported that exposure of the mouse intestine to NMPs induced local inflammation (Li et al. 2020), altered the microbiome (Lu et al. 2018)—particularly favoring facultative pathogenic strains such as *Staphylococcus aureus* triggered metabolic dysfunction (Jin et al. 2019), affected hepatic lipid metabolism (Wang et al. 2019; Lu et al. 2019), and modified host–pathogen interactions (Lu et al. 2019). Similar outcomes were observed by Deng et al., where oral exposure of mice to NMPs led to alterations to the gut microbiome and disruption of serum and liver markers related to amino acid synthesis and metabolism, energy metabolism, and lipid metabolism, followed by hepatic inflammation (Deng et al. 2017; Li et al. 2021). Further pathway analysis in this study confirmed that the expression of inflammatory cytokines is regulated by NF‐κB/iNOS‐mediated oxidative stress. This mechanism was more pronounced in the PS‐Fibers group, which may be associated with the expression of upstream factors (FAK, TLR4).

This study revealed that PS‐Beads and PS‐Fibers activated the NF‐κB/iNOS pathway through distinct upstream molecules (i.e., TLR4 vs. TLR4 + FAK, respectively). Both shapes of PS‐NMPs ultimately induce inflammation and barrier damage via the NF‐κB/iNOS pathway, which aligns with the role of NF‐κB as a central regulator of inflammation. In this study, both shapes of PS‐NMPs significantly increased NF‐κB protein levels (*p* < 0.05), and iNOS expression was positively correlated with NF‐κB activity. Moreover, knockdown of NF‐κB significantly suppressed both the up‐regulation of iNOS and intestinal barrier damage induced by either shape of PS‐NMPs (*p* < 0.01), confirming NF‐κB as the common downstream target in both mechanisms. TLR4, a member of the pattern recognition receptor family, primarily recognizes pathogen‐associated molecular pattern and damage‐associated molecular patterns. A study conducted by Yang et al. with the use of a subacute mouse gavage model, reported that PS‐NMPs promoted peripheral inflammation via activation of the hippocampal TLR4/MyD88/NF‐κB signaling cascade (Yang et al. 2023). Wu et al. demonstrated that PS‐NMPs triggered uterine fibrosis in mice by activating the TLR4/NOX2 axis, leading to oxidative stress (Wu et al. 2022). These findings indicate that TLR4 is a key mediator of toxicity induced by PS‐NMPs. The present study further demonstrated that both PS‐Beads and PS‐Fibers exerted toxic effects on colon tissue via the TLR4/NF‐κB/iNOS signaling pathway, a mechanism that may be associated with their polymer characteristics.

FAK, a nonreceptor tyrosine kinase, plays a critical role in cell adhesion, migration, and mechanotransduction. Liang et al. reported that FAK can be activated by mechanical stimuli such as stretching or piercing, which alter cytoskeletal tension and promote FAK clustering and phosphorylation at focal adhesions (Liang et al. 2023). In this study, exposure to PS‐Fibers significantly increased p‐FAK levels, whereas PS‐Beads had no significant effect on p‐FAK activity. Knockdown of FAK inhibited both NF‐κB activation and the toxic effects induced by PS‐Fibers, suggesting that PS‐Fibers may activate FAK via mechanical stimulation, thereby initiating downstream cascades. Further mechanistic studies have shown that integrin clustering activates FAK, which subsequently promotes NF‐κB phosphorylation via Src family kinases (Shimojo et al. 2015). In the present study, a significant positive correlation was observed between p‐FAK and NF‐κB expression in the PS‐Fibers group, supporting the existence of this signaling axis.

The fiber pathogenicity paradigm (FPP) has established that the pathogenic potential of fibrous materials is largely determined by their physicochemical characteristics, including fiber length, diameter, and bending stiffness (Donaldson et al. 2010; Kane et al. 2018). In particular, fibers longer than 10 μm are generally considered biologically active due to their reduced phagocytosis efficiency (Kane et al. 2018). Therefore, as an initial attempt to investigate the biological effects of NMPs fibers, we selected a fiber length of approximately 10 μm, referencing the dimensional characteristics commonly used in studies of asbestos and carbon nanotubes (Schinwald et al. 2012; Zhu et al. 2016; Manke et al. 2014). Regarding fiber diameter, technical limitations in material synthesis restricted the minimum achievable diameter in our study to approximately 500 nm, which represents the smallest diameter currently attainable for our synthesized PS‐Fibers. Thus, we initially focused on the fiber with a diameter of 500 nm and length of 10 μm. Notably, the studies using PS‐Beads have revealed the particle size–dependent effects of NMPs on the FAK signaling pathway. Previous studies have demonstrated that the intestinal epithelial tight junction barrier damage induced by 5‐μm PS‐Beads was more severe than that induced by 0.2‐μm PS‐Beads and 1‐μm PS‐Beads (Zeng et al. 2024). In future studies, we will further investigate the effects of fibers with different diameters on colon toxicity and their potential regulation of the FAK signaling pathway.

In addition, NMPs fibers differ substantially from asbestos or mineral fibers in their physical properties. They are typically more slender, flexible, and prone to bending, coiling, or entanglement, which prevents them from maintaining the rigid, needle‐like morphology characteristic of asbestos fibers. Importantly, the bending stiffness of fibers could regulate their effective length and directly influence biological effect. Consequently, the pathogenic behavior of NMPs fibers may differ from that of relatively rigid mineral fibers such as asbestos. In future studies, we will further compare the intestinal toxicity induced by fibers with different diameters, lengths, and bending stiffness in order to better clarify how these physical parameters influence their biological effects.

At present, there is limited evidence directly linking asbestos or carbon nanotube exposure to the activation of the FAK signaling pathway. This may be partly due to the fact that most studies on these fibrous materials have primarily focused on respiratory exposure and lung‐related outcomes, rather than intestinal toxicity. In future studies, we will further compare the mechanisms underlying intestinal toxicity induced by fibers with different mechanical properties, particularly between relatively rigid fibers and more flexible polymeric fibers, in order to clarify potential differences in their pathogenic mechanisms.

In this study, a mouse gavage model for subacute exposure and an FHC cell model for acute exposure were employed. The toxicity patterns of PS‐NMPs were consistent across both systems: PS‐Fibers demonstrated stronger toxicity than PS‐Beads, effects were dose‐dependent, and pathway activation was dependent on particle shape. Nevertheless, differences emerged between the in vivo and in vitro models: in animals, digestive fluids and intestinal microbiota may modify the surface properties of PS‐NMPs, whereas in cultured cells, particles interact directly without undergoing biotransformation. Overall, this work established particle shape as a key determinant of the toxicity of PS‐NMPs in mouse colon tissue. These distinct mechanistic profiles advance our understanding of morphological heterogeneity in the toxicity of NMPs and provide a scientific foundation for environmental risk assessment and health protection.

## 5 Conclusion

Exposure to PS‐Fibers or PS‐Beads alone induced inflammation of the mouse colon and compromised the physical, chemical, and immune barriers. These effects included shortening of colon length, mucosal damage, inflammatory cell infiltration, increased intestinal permeability, decreased expression of tight junction proteins and mucins, elevated pro‐inflammatory cytokine levels, and reduced anti‐inflammatory cytokine levels. The detrimental effects of PS‐Fibers were more pronounced than those of PS‐Beads, and the toxicity of these differently shaped PS‐NMPs increased with dose. Both PS‐Fibers and PS‐Beads triggered colonic inflammation and disrupted intestinal barrier function by activating the TLR4/NF‐κB/iNOS signaling pathway. Furthermore, PS‐Fibers also caused additional intestinal barrier impairment via the FAK/NF‐κB/iNOS pathway, resulting in more severe damage than induced by PS‐Beads.

## Author Contributions

**Yuting Li:** conceptualization, investigation, data curation, supervision, writing – original draft. **Shibo Bao:** investigation, visualization, writing‐original draft, writing – review and editing, data curation. **Puyang Huang:** methodology, investigation. **Jiahui Wang:** investigation, methodology, supervision. **Hongfeng Li:** methodology, investigation. **Yahong Liu:** methodology, visualization. **Kai Wang:** methodology, visualization. **Jianjun Du:** methodology, visualization. **Lingjun Yan:** conceptualization, project administration, funding acquisition, supervision. **Guowei Pan:** conceptualization, project administration, funding acquisition, supervision, writing – review and editing. **Wei Sun:** conceptualization, project administration, funding acquisition, supervision, writing – review and editing.

## Funding

This work was supported by the National Natural Science Foundation of China (Grant Number U21A20399) and the Science and Technology Innovation Team Project of China Medical University 2022 (Grant Number CXTD2022006). The funder had no role in study design, data collection and analysis, decision to publish, or preparation of the manuscript.

## Ethics Statement

All animal experiments were performed in strict compliance with the ethical standards established by the Institutional Animal Care and Use Committee of China Medical University (Approval No. CMU20241886), which reviewed and authorized both the experimental protocols and animal handling procedures.

## Conflicts of Interest

The authors declare no conflicts of interest.

## Data Availability

The datasets used and/or analyzed during the current study are available from the corresponding author on reasonable request.
